# Methanol fixation for scanning electron microscopy of plants

**DOI:** 10.1186/s42649-020-00028-5

**Published:** 2020-05-25

**Authors:** Ki Woo Kim

**Affiliations:** 1 School of Ecology and Environmental System, Kyungpook National University, Sangju, 37224 Republic of Korea; 2 Tree Diagnostic Center, Kyungpook National University, Sangju, 37224 South Korea

**Keywords:** Fixation, Glutaraldehyde, Methanol

## Abstract

Plant specimens for scanning electron microscopy (SEM) are commonly treated using standard protocols. Conventional fixatives consist of toxic chemicals such as glutaraldehyde, paraformaldehyde, and osmium tetroxide. In 1996, methanol fixation was reported as a rapid alternative to the standard protocols. If specimens are immersed in methanol for 30 s or longer and critical-point dried, they appear to be comparable in preservation quality to those treated with the chemical fixatives. A modified version that consists of methanol fixation and ethanol dehydration was effective at preserving the tissue morphology and dimensions. These solvent-based fixation and dehydration protocols are regarded as rapid and simple alternatives to standard protocols for SEM of plants.

## Introduction

Plant specimens must be fixed for SEM because they cannot withstand water removal by the vacuum system without distortion (Pathan et al. 2010). A standard protocol for SEM of plant specimens comprises a series of procedures: chemical fixation, dehydration, critical point drying, and metal coating (Yuan et al. 2020). The chemical fixation involves immersing specimens in solvents such as glutaraldehyde, paraformaldehyde, and osmium tetroxide for various periods (Chieco et al. 2012). Depending on the specimen dimension, this protocol usually takes several hours (Neinhuis and Edelmann 1996). Meanwhile, dried materials such as seeds, wood blocks, and herbarium specimens are metal coated for SEM observations without any other preparations. Using either variable pressure or low-temperature SEM also reduces labor and time for specimen preparations (Kim 2013; Talbot and White 2013b).

A well-known artefact of preparing biological specimens for SEM is the tissue shrinkage (up to 75% of their original size) during fixation, dehydration, and critical point drying steps (Talbot and White 2013a). It is necessary to find a better fixative that allows morphological preservation with reduced labor and time. Another property of a good fixative represents the possession of a modest toxicological and flammability profile that permits the safe use of the chemical (Eltoum et al. 2001). This review aims to provide insights and technical guidance about the methanol fixation as an alternative to standard protocols for SEM of plants.

## Methanol fixation

Methanol (CH_3_OH) is the simplest alcohol and highly polar solvent. It is closer to the structure of water than ethanol (C_2_H_5_OH) and rapidly penetrates tissues, simply replacing free water throughout the tissues (Eltoum et al. 2001; Talbot and White 2013a). As traditional coagulant fixatives of dehydrant types, methanol and ethanol maintain tissue morphology at the light microscopic level fairly well (Eltoum et al. 2001). Methanol was used to fix the tension-stressed cell wall length dimensions of rye coleoptile segments, which might be due to the removal of water from the cell walls and thereby increased hydrogen bonding between cell wall polymers (Edelmann 1995).

**Fig. 1:**
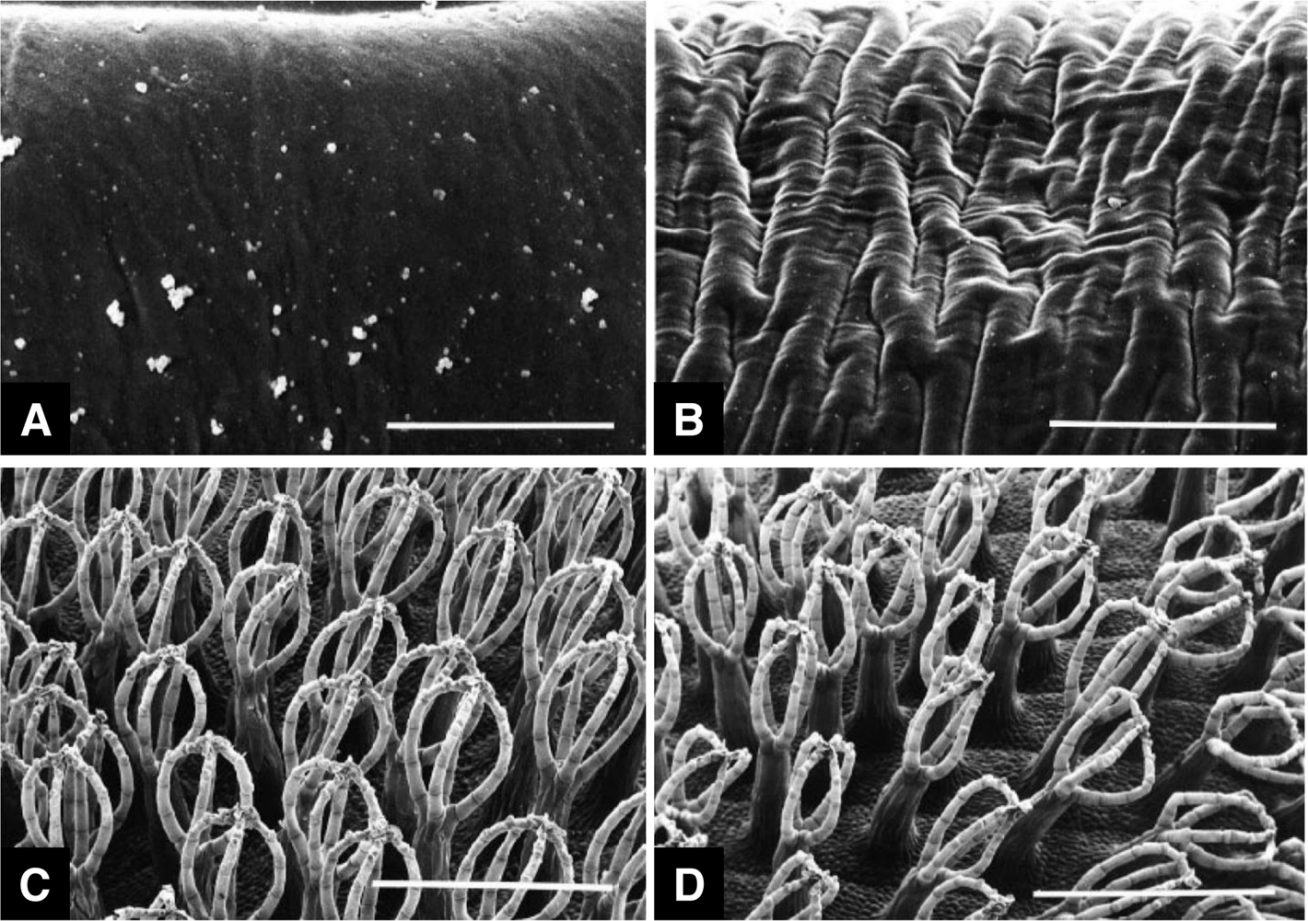
Scanning electron micrographs of leaves. (Left) Specimens fixed and dehydrated in methanol followed by critical point drying. (Right) Specimens treated with a conventional protocol. **a** and **b** Rye coleoptiles. Bars = 5 μm. **c** and **d***Salvinia auriculata* trichomes. Bars = 1 mm. From Neinhuis and Edelmann 1996 with permission from the publisher

In 1996, Neinhuis and Edelmann in Germany first reported the practical use of methanol as a fixative of plant surfaces for SEM. They immersed plant specimens in methanol for 30 s or longer and dried them using a critical point drier (Table 1). For comparison, they employed the standard protocol for SEM. The methanol fixation/dehydration protocol revealed overall smooth epidermis of rye coleoptiles (Fig. 1a). Meanwhile, a regular folding due to shrinking was observed in the standard protocol treatment (Fig. 1b). *Salvinia auriculata* trichomes were assumed to be better preserved using the methanol fixation/dehydration protocol than those treated with the standard protocol (Fig. 1c and d). Taken together, the methanol incubation for fixation/dehydration was proposed as a suitable treatment prior to critical point drying of plant specimens (Neinhuis and Edelmann 1996). Owing to this instant fixation, specimen shrinkage would be prevented, resulting in an improved preservation of cell dimensions (Zelko et al. 2012). With regard to plant epicuticular waxes, the use of methanol as a fixative has been considered as desirable since methanol is relatively less damaging to plant waxes as compared to ethanol or acetone (Pathan et al. 2010).

**Table 1:** Two methanol fixation protocols for scanning electron microscopy of plant specimens

Methanol fixation^a^	Methanol fixation – ethanol dehydration^b^
Immersion in 100% methanol for 30 s	Fixation in 100% methanol for 10 min
	Dehydration in 100% ethanol for 30 min
	(two times)
Critical point drying	Critical point drying

^a^from Neinhuis and Edelmann 1996

^b^from Talbot and White 2013a

## Ethanol fixation

Another solvent-based fixation was proposed to reduce the health hazards of formalin-based fixatives. Ethanol is also a widely used fixative that removes water and coagulates proteins in tissues (Eltoum et al. 2001). Commercialized as FineFIX™, an ethanol-based fixative for SEM does not contain formalin (Chieco et al. 2012). Plant leaves and trichomes treated with the ethanol-based fixative were comparable to those treated with the standard fixative in morphological preservation.

## Examples of methanol fixation since 1996

### Leaf stomata

**Fig. 2:**
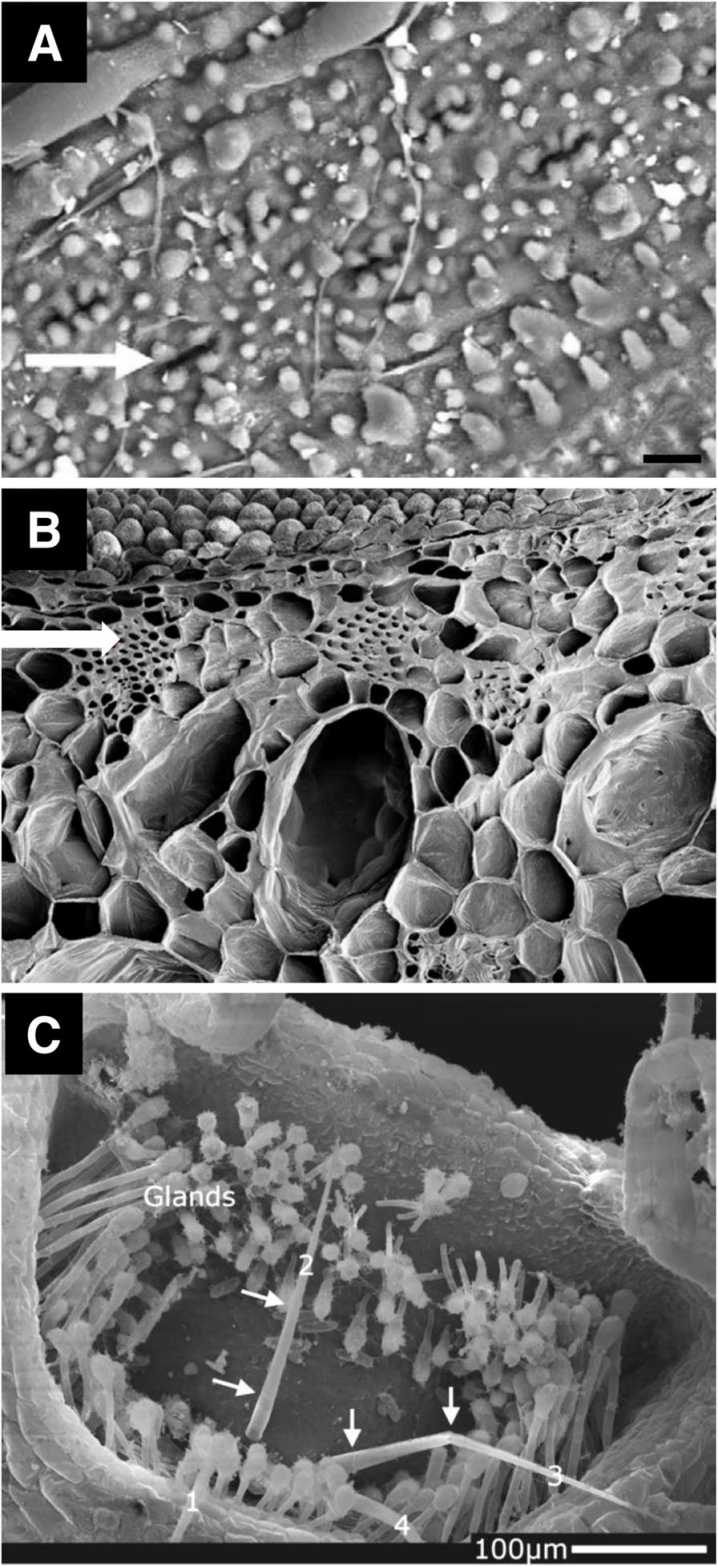
Scanning electron micrographs of methanol-fixed plants. **a** Rice leaf. Arrow = stoma. Bar = 10 μm. **b** Cross section of *Colocasia fallax* leaf fixed in methanol. Arrow = sclerenchyma. **c** Suction trapdoor of a carnivorous plant *Utricularia australis*. Note four trigger hairs (1–4). Arrows = cell-to-cell junctions. A: from Das and Baruah 2008; B: from Sacher et al. 2019; C: from Poppinga et al. 2017 with permission from the publisher

Leaf and stem specimens of rice were fixed and dehydrated with methanol, and critical-point dried (Das and Baruah 2008). Stomatal frequencies (number of stomata mm^− 2^) were measured from the methanol-fixed leaf specimens (Fig. 2a). The morphological preservation was sufficient for the xylem observation in stems.

### Leaf sclerenchyma

Cross sections from peltate-leaf plant *Colocasia fallax* were dissected using a razor blade (Sacher et al. 2019). They were fixed in methanol and critical-point dried. Sclerenchyma could be distinguished from other tissues in the methanol-fixed cross sectioned leaf specimens (Fig. 2b).

### Carnivorous trap

An aquatic carnivorous plant *Utricularia australis* has suction traps for animal capturing. The trap was fixed in methanol and critical-point dried (Poppinga et al. 2017). Glands and trigger hairs were found in the trapdoor (Fig. 2c). Cell-to-cell junctions could be discernable on the trigger hairs.

### Fruit skin

The integrity of tomato fruit was investigated using SEM (Bargel and Neinhuis 2005). Cross sections of fruit were fixed in methanol for 1 min and critical-point dried. The cuticles and epidermal cell walls could be identified at different ripening stages.

## Variations of methanol fixation

**Fig. 3:**
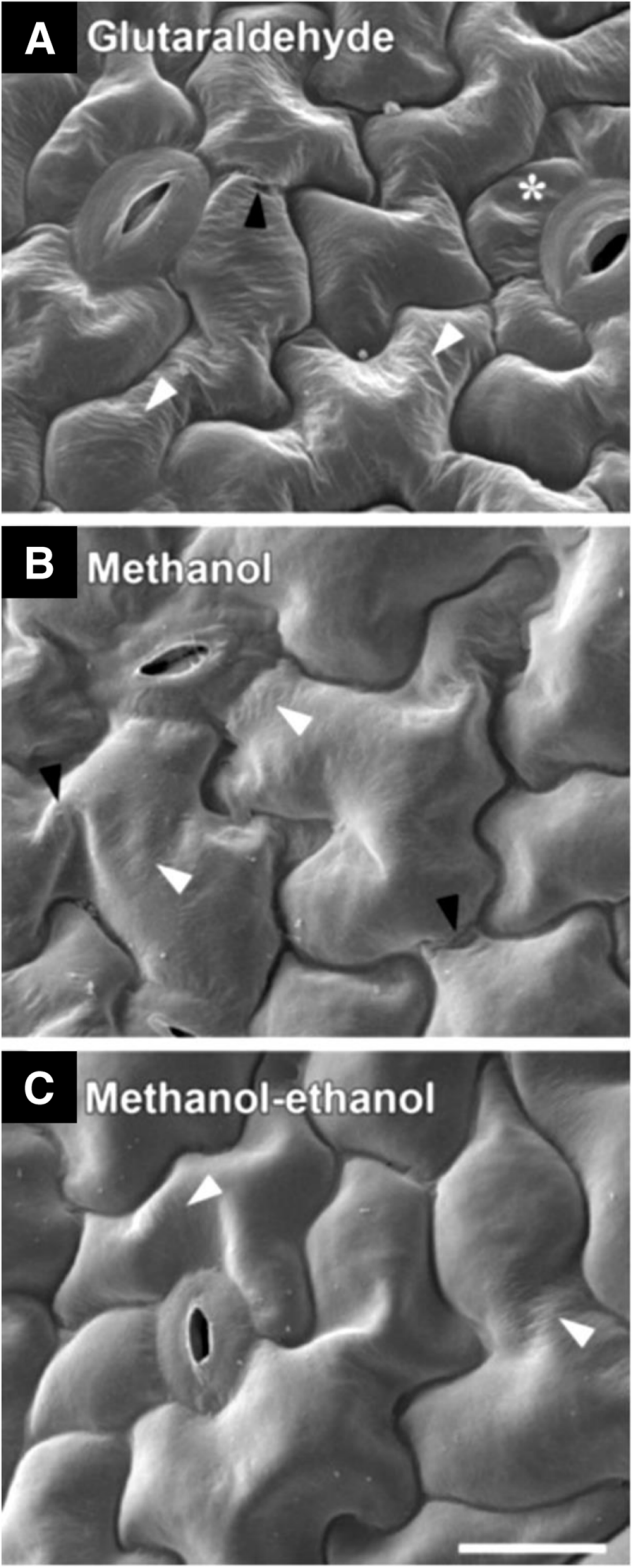
Scanning electron micrographs of *Arabidopsis thaliana* leaves. **a** Specimen fixed in glutaraldehyde. **b** Specimen fixed in methanol followed by critical point drying in methanol. **c** Specimen fixed in methanol followed by ethanol dehydration and critical point drying in ethanol. Star = partial cell collapse. Black arrowhead = cell wall folding. White arrowhead = cell wall wrinkle. Bar = 20 μm. From Talbot and White 2013a with permission from the publisher

In 2013, Talbot and White in Australia proposed another version of the methanol fixation (Table 1). They modified the original methanol fixation protocol to preserve the dimensions of critical point-dried specimens. Leaves of *Arabidopsis thaliana* were (i) fixed in 3% glutaraldehyde overnight at 4 °C, (ii) fixed in methanol for 10 min followed by methanol dehydration and critical point drying with methanol, and (iii) fixed in methanol for 10 min followed by ethanol dehydration and critical point drying with ethanol. Overall, the epidermis treated with the standard fixative (Fig. 3a) was not well fixed as those with the solvent-based protocols (Fig. 3b and c) in preservation quality. The methanol fixation followed by ethanol dehydration and critical point drying with ethanol resulted in the least cell wall wrinkling with negligible cell collapse or cell wall folding (Talbot and White 2013a).

As another example, forage grass roots were fixed in methanol at room temperature overnight and dehydrated with 100% ethanol three times (Saleh et al. 2019). SEM revealed roots and their endophytic bacteria in the rhizosphere. Root hairs, rod-shaped bacteria, and bacterial aggregates could be resolved.

**Fig. 4:**
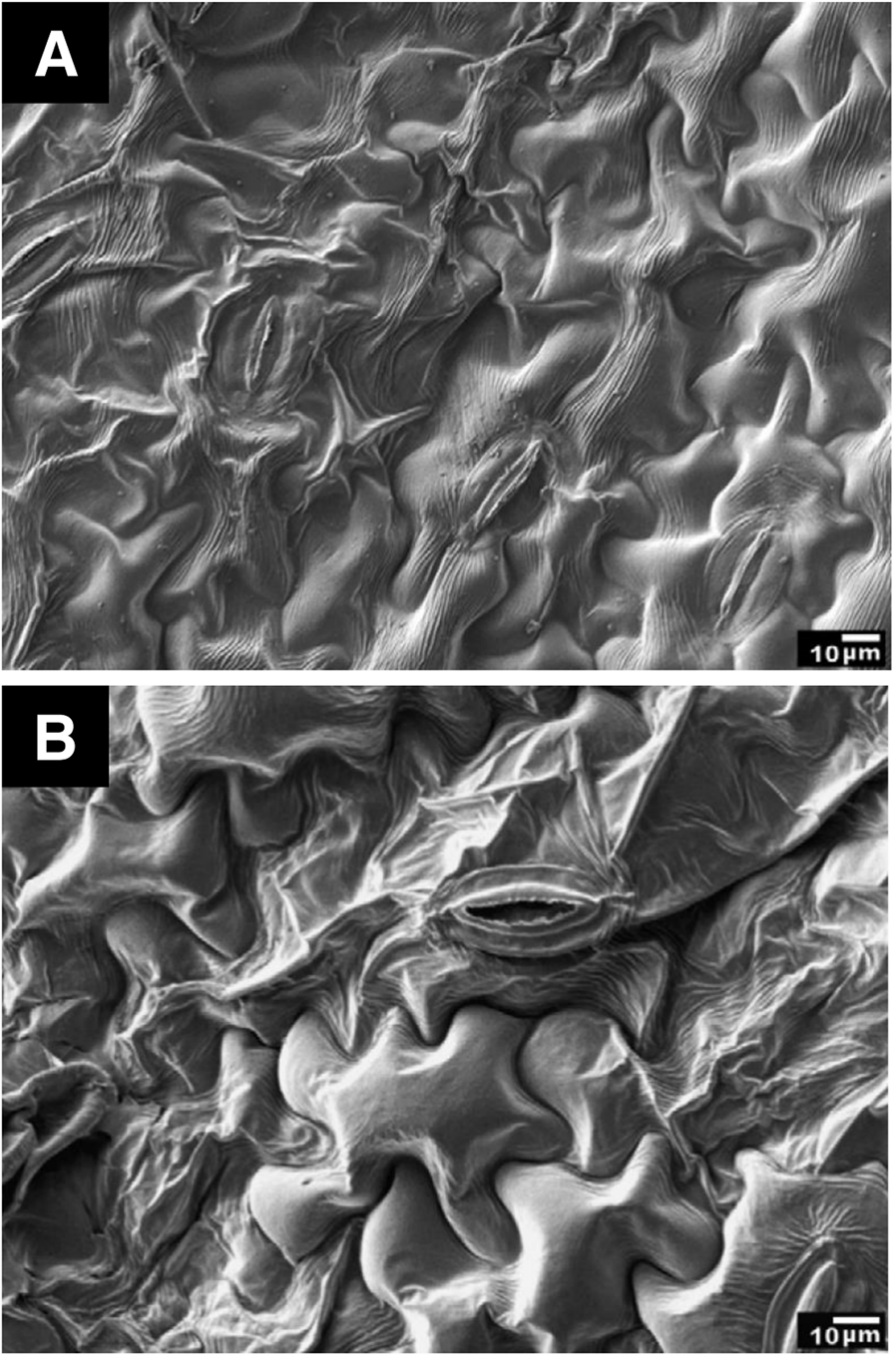
Scanning electron micrographs of bean leaves. **a** Specimen fixed in methanol for 20—40 s followed by air-drying. **b** Specimen fixed in liquid nitrogen-cooled methanol for 20—40 s and liquid nitrogen followed by freeze-drying. Bars = 10 μm. From Pathan et al. 2010 with permission from the publisher

The variation of methanol fixation was also tested with bean leaves. The leaves were (i) fixed in methanol for 20—40 s followed by air-drying, and (ii) fixed in liquid nitrogen-cooled methanol for 20—40 s and liquid nitrogen followed by freeze-drying (Pathan et al. 2010). Both protocols exhibited partial preservation of leaf epidermis (Fig. 4). The latter protocol was evaluated as better than the former one in preservation quality. These findings indicate the importance of a drying procedure even after methanol fixation.

## Methanol fixation for fluorescence microscopy

**Fig. 5:**
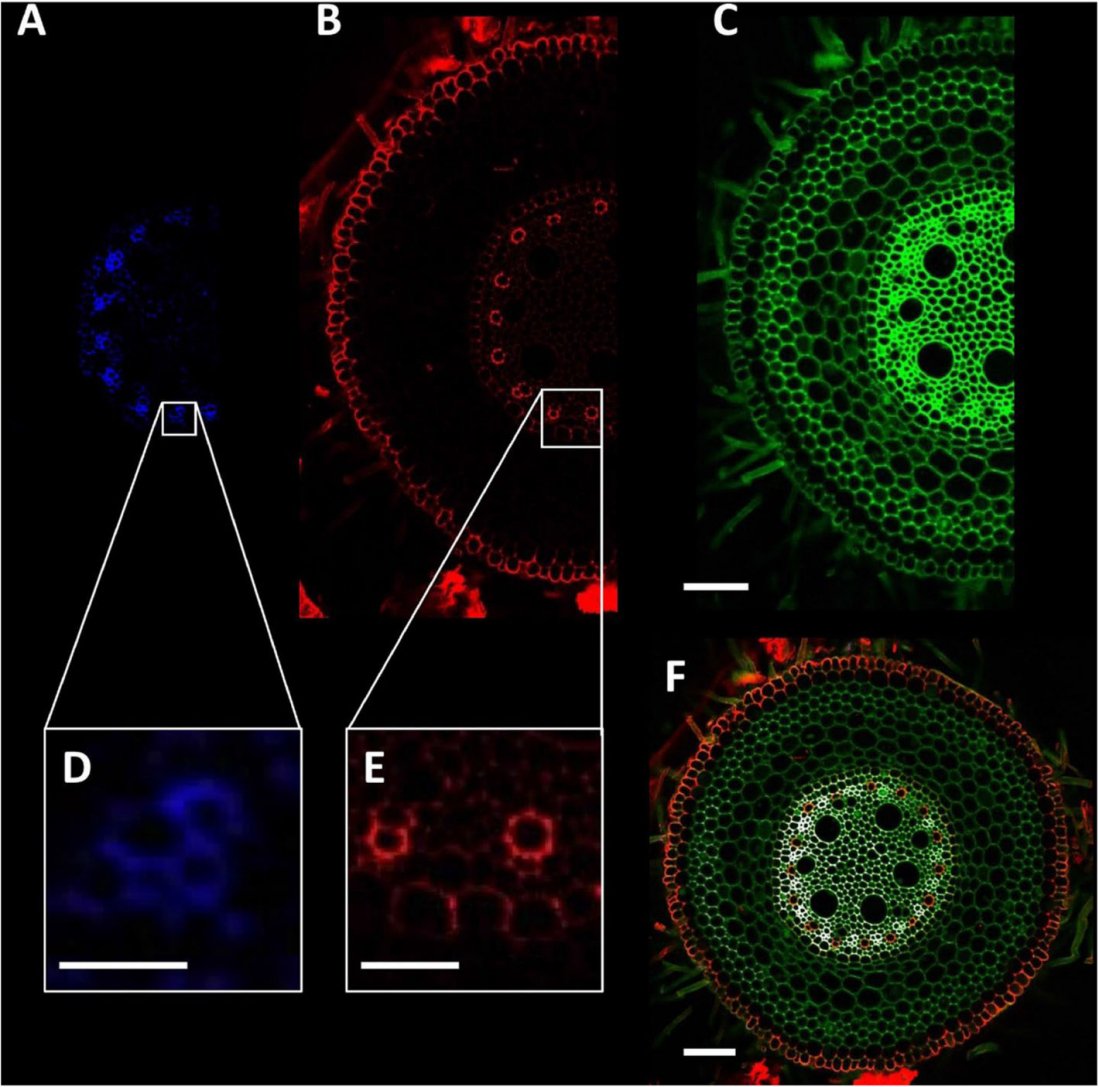
Confocal laser scanning micrographs of wheat root cells. **a** and **d** Phloem. **b** Autofluorescence signal. **c** Total cell wall image. **e** Xylem. **f** Composite image of (**a**), (**b**), and (**c**). Bars = 100 μm (**a-c**, **f**), 25 μm (**d**), and 50 μm (**e**). From Atkinson and Wells 2017 with permission from the publisher

Plant specimens are fixed and stored in methanol for the high-throughput quantification of cell and tissue structures (Atkinson and Wells 2017). Root anatomical features including phloem, xylem, epidermis, and endodermis were observed in the methanol-fixed wheat (Fig. 5). Methanol fixation preserved root elasticity and did not influence fluorescent staining of root specimens, which was different from the other fixatives (Zelko et al. 2012).

## Cautions

The use of methanol as a single fixative is not always effective at preserving cell morphology. In rat liver cells, methanol was evaluated for its performance as a fixative of cytoskeletal components for immunofluorescence. However, such a methanol-based precipitation fixation was not suitable, as it caused numerous artefacts due to cell shrinkage (Vielkind and Swierenga 1989).

Human breast cancer cells were fixed in methanol at − 20 °C for 10 min and dehydrated in ethanol and critical-point dried. The methanol fixation resulted in poor preservation of plasma membrane integrity (Hoetelmans et al. 2001). The authors objected to the use of methanol as a single fixative in the case of human breast cancer cells. In general, aldehyde fixation protocols perform significantly better than organic solvents with less severe loss of biochemical information in animals (Hobro and Smith 2017). These findings suggest that methanol fixation may be effective only in plants.

## Conclusions

Methanol fixation was developed as an alternative to conventional protocols for SEM observations of plants. Immersion in methanol for 10 min and critical point drying were appropriate to preserve the tissue morphology of various plants. Simultaneous fixation and dehydration in methanol has been recognized as a rapid alternative to standard aldehyde-based fixatives and solvent-based dehydration steps. A modified version was developed to include methanol fixation and ethanol dehydration, which took longer for SEM observations than the original protocol. However, it was regarded to provide a better tissue preservation than the original protocol. This solvent-based fixation and dehydration does not necessitate the use of toxic chemicals such as glutaraldehyde, paraformaldehyde, and osmium tetroxide. These findings suggest that the methanol fixation and ethanol dehydration may be used widely to a variety of plant specimens.

## Data Availability

Data and materials available on request.

## Abbreviation

SEM: Scanning electron microscopy
